# Notice

**Published:** 1901-08

**Authors:** 


### NOTICE.

AT a recent meeting of the Board of Trustees of the State Sanitarium, in Milledgeville, July 19, 1901, a commission of physicians, consisting of Dr. W. S. Elkin, Dr. James B. Baird and Dr. J. B. S. Holmes, was appointed to conduct a competitive examination, in Atlanta, Monday, October 7, 1901, of applicants for appointment on the medical staff of the Sanitarium.

This examination, required by the laws of the State, is necessary to render applicants eligible for election to any vacancy that may occur on the medical staff, but does not apply to persons now holding office in the Sanitarium.

All applications must be filed with the chairman of the commission, Dr. W. S. Elkin, Atlanta, at least one day before the time set for the examination.

Any member of the commission will cheerfully furnish, upon request, all information relating to the examination that may be desired by any applicant.

---

SECTARIANISM in medicine has always been a general scandal and the subject of much satirical comment on the part of the lay public. But there is at present a decided tendency on the part of the medical profession to obliteration of school distinctions, which means a union of class interests and a disappearance of prejudice. From what direction the approach has come is not evident, but we believe that the disappearance of homeopathy from among the homeopaths has removed the element of repulsion and attraction has thus become in a measure involuntary. It is very certain by way of proving this proposition that the regular school has not adopted homeopathic principles and methods. The sentiment that if a beast be lord of beasts his crib shall stand at the king's mess has always animated a large number of the so-called regular profession and they stand ready and willing to adopt that which is good whatever its origin. If this constitutes the basis of fraternal union, there will be no complaint from any quarter.

